# Effect of Smoking on the Development of Migraine in Women: Nationwide Cohort Study in South Korea

**DOI:** 10.2196/58105

**Published:** 2024-08-23

**Authors:** Seung Ae Kim, Kyungdo Han, Soyoun Choi, Michelle Sojung Youn, Hyemin Jang, Mi Ji Lee

**Affiliations:** 1 Department of Neurology, Seoul National University Hospital, Seoul National University College of Medicine, Seoul, Republic of Korea; 2 Department of Translational Medicine, Seoul National University College of Medicine, Seoul, Republic of Korea; 3 Department of Statistics and Actuarial Science, Soongsil University, Seoul, Republic of Korea; 4 Department of Neurology, Seoul Hospital, Ewha Womans University College of Medicine, Seoul, Republic of Korea; 5 Department of Neurology, Nowon Eulji Medical Center, Eulji University School of Medicine, Seoul, Republic of Korea

**Keywords:** smoking, migraine, premenopausal women, postmenopausal women, incidence, risk

## Abstract

**Background:**

Smoking is known to be a significant risk factor for various diseases. Migraine, a condition requiring careful lifestyle management, currently lacks specific guidelines advocating for smoking cessation as a preventive measure. Although cross-sectional studies have suggested a potential link between smoking and an increased risk of migraine, the findings have been inconsistent and conflicting. To date, there has been no longitudinal study which investigated the effect of smoking on the risk of migraine in a prospective setting.

**Objective:**

This longitudinal study aimed to investigate the impact of smoking on the incidence of migraine in women and examine the modifying effect of menopausal status.

**Methods:**

Using nationally representative National Health Insurance Service (NHIS) data, women aged ≥40 years who participated in national breast cancer screening in 2009 were followed-up until the end of 2019. Baseline data on smoking status (non-, ex-, and current smoker) as well as the duration and amount of cigarette smoking were collected. A Cox proportional hazards regression model was used to examine the independent effect of smoking on the risk of incident migraine after adjusting for demographics, comorbidities, and female reproductive factors. The results were stratified by menopausal status, and an interaction analysis (smoking × menopause) was performed.

**Results:**

In total, 1,827,129 women were included in the analysis. Women with a history of smoking exhibited a higher risk of developing migraine, compared with nonsmokers. Specifically, a higher risk of migraine was observed in women with past (adjusted hazard ratio [HR] 1.044, 95% CI 1.000-1.089) and current cigarette use (adjusted HR 1.050, 95% CI, 1.023-1.079) than in nonsmokers. The effect was greater in premenopausal women (adjusted HR 1.140, 95% CI, 1.108-1.172) than in postmenopausal women (adjusted HR 1.045, 95% CI 1.018-1.073*; P*<.001). The risk increased with an increased amount of smoking, with a greater association in premenopausal women (*P*<.001).

**Conclusions:**

Smoking increases the risk of migraine in women, with a dose-dependent relationship. Menopause modifies this effect. Our findings suggest that smoking is an important modifiable risk factor of migraine, with a higher impact in premenopausal women. The interaction between smoking and estrogen may increase the vulnerability of the migraine brain.

## Introduction

Migraine is a prevalent and disabling disorder, ranking among the top causes of years lived with disability according to the Global Burden of Disease Study [1]. Migraine affects over 1 billion people globally, significantly diminishing patients’ quality of life and leading to considerable economic consequences due to health care costs and lost productivity [2,3]. The burden of migraine is progressively increasing worldwide [4]. As migraine imposes a significant toll on patient quality of life and daily function, the identification of modifiable risk factors becomes imperative for effective management and prevention [5].

Various factors, such as lifestyle, hormonal changes, environmental influences, and medications can significantly impact the initiation, duration, and intensity of migraine [6]. Identifying the specific factors linked to migraine episodes offers valuable insights to devise preventive measures and effectively manage triggers associated with migraine [6,7].

Smoking is known to be a significant risk factor for various diseases [8-10]. Despite the importance of lifestyle management in mitigating migraine, there are currently no specific guidelines advocating for smoking cessation as a preventive measure for migraine. Although cross-sectional studies have suggested a potential link between smoking and an increased risk of migraine, the findings have been inconsistent and conflicting. Although some studies suggest a higher occurrence of migraine in smokers and indicate that smoking may influence the activity of the disease [11-13], others propose no significant correlation between smoking and migraine [14-16]. However, as these results were derived from cross-sectional studies, possible biases, such as behavioral modifications due to migraine, cannot be excluded, making it difficult to establish a causal relationship.

Thus, prospective longitudinal research is essential to establish a definitive causal relationship between smoking and migraine. We, therefore, aimed to conduct a population-based prospective cohort study to investigate the effects of smoking on the incidence of migraine in women. The effects of smoking were further stratified according to hormonal status to determine the differential risk and control for potential confounders.

## Methods

### Data Source

In South Korea, adults are required to undergo regular national health examinations, typically conducted every 2 years for the general population, with annual screenings available for individuals engaged in physical labor [17]. These health assessments are documented in the Korean health examination (KHE) database, which functions as a comprehensive repository of health-related information. The KHE database captures a broad array of data including age, household income, and the history of common diseases such as diabetes mellitus, hypertension, dyslipidemia, ischemic heart disease, and chronic kidney disease. Additionally, health-related behaviors such as alcohol consumption, smoking, and regular physical activity are recorded. For women, specific reproductive health data, including age at menarche, age at menopause, parity, history of hysterectomy, breastfeeding history, and duration of hormone replacement therapy, are typically documented and maintained every 2 years in the National Cancer Screening Program database [18].

The NHIS program provides medical services to the entire Korean population, resulting in a substantial sample size of over 50 million individuals [19]. The NHIS database encompasses comprehensive records of all claims made under the national health insurance program and categorizes diagnoses using the *International Classification of Diseases, Tenth Revision* (*ICD-10*) codes [20].

### Study Population and Design

To examine the impact of smoking on migraine development in women, we combined data from the KHE and NHIS databases. This allowed us to longitudinally assess the occurrence of migraine by capturing NHIS claims according to baseline demographics, health profiles, and female reproductive factors recorded in the KHE.

Our study included women who met the following criteria: (1) aged 40 years or older, and (2) participation in the breast or vaginal cancer screening program conducted by the KHE. This age specification aligns with the initiation of cancer screenings in Korea, which begins at the age of 40 years. The exclusion criteria included (1) a history of hysterectomy, (2) claims of migraine during the washout period (from 2002 to 2009), and (3) missing baseline characteristics or female hormonal factors.

### Exposure Variable

The primary exposure variable studied was cigarette smoking. Participants were categorized into three groups: (1) nonsmokers, comprising individuals who had smoked fewer than five packs (100 cigarettes in total or five packs containing 20 cigarettes per pack) in their lifetime; (2) ex-smokers, encompassing women with prior cigarette use who had smoked more than five packs in their lifetime but were not actively smoking during the investigation; and (3) current smokers, including women with ongoing cigarette use who had consumed more than five packs in their lifetime and were still smoking at the time of the investigation. Pack-years were calculated by multiplying the smoking years by the average daily cigarette consumption and dividing by 20.

### Covariates

As covariates, we assessed menopause status and additional factors. Participants were stratified into premenopausal and postmenopausal women according to spontaneous menopause as recorded in the KHE questionnaire, after excluding those who reported a history of hysterectomy. Data on age, income, alcohol consumption status, exercise, diabetes mellitus, hypertension, dyslipidemia, chronic kidney disease, BMI, and waist circumference were recorded. Diabetes mellitus was defined as having a fasting glucose level of 126 mg/dL or higher or being on diabetes medications with an *ICD-10* code of E11-E14. Hypertension was defined as having a systolic blood pressure of 140 mmHg or higher, a diastolic blood pressure of 90 mmHg or higher, or being on hypertension medications with *ICD-10* codes of I10-I13 or I15. Dyslipidemia was defined as having a total cholesterol level of 240 mg/dL or higher or being on dyslipidemia medications with an *ICD-10* code of E78. Chronic kidney disease was defined as having an estimated glomerular filtration rate of less than 60 mL/min/1.73 m². Data on female reproductive factors, such as age at menarche, parity, breastfeeding, and oral contraceptive use, and duration, were also collected for all participants. Postmenopausal women were asked about their response to hormone replacement therapy and their age at menopause.

### Outcome Assessment

Migraine incidence was determined by capturing the *ICD-10* code G43 in the NHIS claims during the observation period. We implemented a washout period from 2002 to 2009 during which patients diagnosed with G43 were excluded from this study. Additionally, patients diagnosed within 1 year of the lag period were excluded.

### Ethical Considerations

This study was conducted in accordance with the principles stated in the Declaration of Helsinki and was approved by the Institutional Review Board of the Soongsil University (SSU-202007-HR-236‐01). In this study, the necessity to obtain informed consent was waived owing to the use of anonymized archival data.

### Statistical Analysis

The incidence rate was computed as 1000 person-years for each group, followed by a hazard ratio (HR) analysis using Cox proportional hazard regression models. The 95% CI was determined for each factor. Continuous variables are represented as the mean and SD using ANOVA. Categorical variables were presented as the number and percentage and were analyzed using the *χ*^2^ test. To address potential confounding variables, we developed a multivariable model to adjust for baseline imbalance between groups, considering age, income, BMI, drinking habits, regular exercise, diabetes mellitus, hypertension, dyslipidemia, chronic kidney disease, age at menarche, parity, breastfeeding history, and oral contraceptive use. We conducted a stratified analysis based on menopausal status and examined the interaction between smoking and menopause in relation to the risk of migraine. To address missing data, we used complete case analysis, which involved excluding observations with any missing data from the analysis.

## Results

### Demographics and Baseline Characteristics

**Figure 1.**
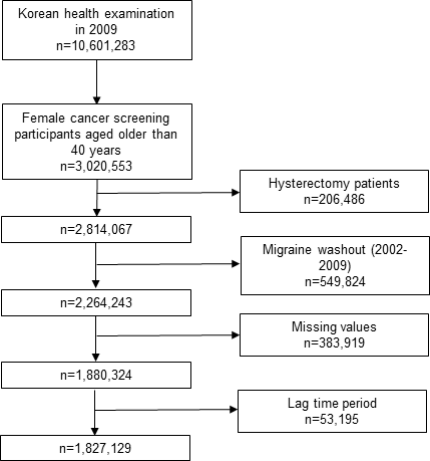
Flowchart of this study’s design showing the selection process of participants from the Korean health examination 2009 dataset. This includes the initial 10,601,283 participants, those who underwent cancer screening and provided data on female reproductive factors, and the final sample of 1,827,129 women older than 40 years after exclusions.

Among 10,601,283 individuals who participated in the KHE in 2009, 5,479,852 who underwent cancer screening provided data on female reproductive factors. Among them, 3,109,100 women older than 40 years were eligible for this study. After removing participants with incomplete data and those with a history of hysterectomy or migraine during the washout or lagging periods, 1,827,129 women were included (Figure 1).

Significant differences were observed in all demographic, social, and lifestyle characteristics, and comorbidities based on smoking status among premenopausal and postmenopausal women (Table 1). Additionally, female reproductive factors, such as age at menarche, number of childbirths, breastfeeding duration, and oral contraceptive use and duration showed significant differences according to smoking status.

When the data were stratified by menopause, significant differences were observed across all demographic, social, and lifestyle characteristics, comorbidities, and female reproductive factors according to smoking status in premenopausal (Table 2) and postmenopausal women (Table 3).

**Table 1.** Baseline demographics and profiles of this study’s participants (women aged ≥40 years from the Korean health examination 2009, followed up until 2019).

		Nonsmoker (n=1,751,873)	Ex-smoker (n=21,674)	Current smoker (n=53,582)	*P* value
Age (years), mean (SD)		54.7 (10.74)	51.9 (11.22)	53 (10.94)	<.001
Income, lowest Q1, n (%)		399,233 (22.8)	5143 (23.7)	14,922 (27.9)	<.001
**Alcohol consumption, n (%)**					<.001
	None	1,436,863 (82)	11,635 (53.7)	25,693 (48)	
	Mild	305,596 (17.4)	9207 (42.5)	23,833 (44.5)	
	Heavy	9414 (0.5)	832 (3.8)	4056 (7.6)	
Regular exercise, n (%)		315,882 (18)	4343 (20)	8367 (15.6)	<.001
Diabetes mellitus, n (%)		157,397 (9)	1869 (8.6)	5216 (9.7)	<.001
Hypertension, n (%)		553,368 (31.6)	5674 (26.2)	14,585 (27.2)	<.001
Dyslipidemia, n (%)		399,005 (22.8)	4946 (22.8)	12,673 (23.7)	<.001
Chronic kidney disease, n (%)		150,789 (8.6)	1793 (8.3)	4455 (8.3)	.01
BMI (kg/m^2^), mean (SD)		23.77 (3.15)	23.47 (3.32)	23.24 (3.37)	<.001
Age at menarche (years), mean (SD)		15.87 (1.98)	15.4 (2.03)	15.85 (2.03)	<.001
**Parity, n (%)**					<.001
	0	42,050 (2.4)	2310 (10.7)	4785 (8.9)	
	1	154,044 (8.8)	4566 (21.1)	10,459 (19.5)	
	≥2	1,555,779 (88.8)	14,798 (68.3)	38,338 (71.6)	
**Breast feeding, n (%)**					<.001
	None	195,614 (11.2)	5183 (23.9)	11,951 (22.3)	
	<6 months	246,012 (14)	4262 (19.7)	8241 (15.4)	
	<12 months	370,819 (21.2)	4165 (19.2)	9396 (17.5)	
	≥12 months	939,428 (53.6)	8064 (37.2)	23,994 (44.8)	
**Oral contraceptive use, n (%)**					<.001
	None	1,503,315 (85.8)	16,102 (74.3)	40,957 (76.4)	
	<1 year	163,311 (9.3)	3316 (15.3)	7504 (14)	
	≥1 year	85,247 (4.9)	2256 (10.4)	121 (9.6)	

**Table 2.** Demographics and characteristics of premenopausal women aged ≥40 years from the Korean health examination 2009 cohort according to smoking status.

		Nonsmoker (n=742,459)	Ex-smoker (n=11,503)	Current smoker (n=25,876)	*P* value
Age (years), mean (SD)		45.11 (4.25)	43.83 (4.03)	44.54 (3.95)	<.001
Income, lowest Q1, n (%)		183,702 (24.7)	2571 (22.4)	7010 (27.09)	<.001
**Alcohol consumption, n (%)**					<.001
	None	544,089 (73.3)	5021 (43.7)	8722 (33.7)	
	Mild	192,706 (26)	5912 (51.4)	14,443 (55.8)	
	Heavy	5664 (0.8)	570 (5)	2711 (10.5)	
Regular exercise, n (%)		128,951 (17.4)	2119 (18.4)	4054 (15.7)	<.001
Diabetes mellitus, n (%)		26,113 (3.5)	392 (3.4)	1238 (4.8)	<.001
Hypertension, n (%)		99,123 (13.4)	1304 (11.3)	3591 (13.9)	<.001
Dyslipidemia, n (%)		77,080 (10.4)	1170 (10.2)	3202 (12.4)	<.001
Chronic kidney disease, n (%)		31,535 (4.3)	451 (4)	1032 (4)	.03
BMI (kg/m^2^), mean (SD)		23.21 (3.05)	22.98 (3.21)	22.94 (3.29)	<.001
Age at menarche (years), mean (SD)		15.1 (1.85)	14.75 (1.86)	15.22 (1.93)	<.001
**Parity, n (%)**					<.001
	0	25,646 (3.5)	1606 (14)	3153 (12.2)	
	1	95,550 (12.9)	2957 (25.7)	6476 (25)	
	≥2	621,263 (83.7)	6940 (60.3)	16,247 (62.8)	
**Breast feeding, n (%)**					<.001
	None	131,348 (17.7)	3548 (30.8)	7987 (30.9)	
	<6 months	181,910 (24.5)	3169 (27.6)	6140 (23.7)	
	<12 months	197,913 (26.7)	2346 (20.4)	5141 (19.9)	
	≥12 months	231,288 (31.2)	2440 (21.2)	6608 (25.5)	
**Oral contraceptive use, n (%)**					<.001
	None	651,150 (87.7)	8288 (72.1)	18,471 (71.4)	
	<1 year	68,573 (9.2)	2054 (17.9)	4574 (17.7)	
	≥1 year	22,736 (3.1)	1161 (10.1)	2831 (11)	

**Table 3.** Demographics and characteristics of postmenopausal women aged ≥40 years from the Korean health examination 2009 cohort according to smoking status.

		Nonsmoker (n=1,009,414)	Ex-smoker (n=10,171)	Current smoker (n=27,706)	*P* value
Age (years), mean (SD)		61.67 (8.41)	60.93 (9.72)	60.82 (9.42)	<.001
Income, lowest Q1, n (%)		215,531 (21.4)	2572 (25.3)	7912 (28.56)	<.001
**Alcohol consumption, n (%)**					<.001
	None	892,774 (88.4)	6614 (65)	16,971 (61.3)	
	Mild	112,890 (11.2)	3295 (32.4)	9390 (33.9)	
	Heavy	3750 (0.4)	262 (2.6)	1345 (4.9)	
Regular exercise, n (%)		186,931 (18.5)	2224 (21.9)	4313 (15.6)	<.001
Diabetes mellitus, n (%)		131,284 (13)	1477 (14.5)	3978 (14.4)	<.001
Hypertension, n (%)		454,245 (45)	4370 (43)	10,994 (39.7)	<.001
Dyslipidemia, n (%)		321,925 (31.9)	3776 (37.1)	9471 (34.2)	<.001
Chronic kidney disease, n (%)		119,254 (11.8)	1342 (13.2)	3423 (12.4)	<.001
BMI (kg/m^2^), mean (SD)		24.19 (3.15)	24.02 (3.36)	23.52 (3.42)	<.001
**Parity, n (%)**					<.001
	0	16,404 (1.6)	704 (6.9)	1632 (5.9)	
	1	58,494 (5.8)	1609 (15.8)	3983 (14.4)	
	≥2	934,516 (92.6)	7858 (77.3)	22,091 (79.7)	
**Breast feeding, n (%)**					<.001
	None	64,266 (6.4)	1635 (16.1)	3964 (14.3)	
	<6 months	64,102 (6.4)	1093 (10.8)	2101 (7.6)	
	<12 months	172,906 (17.1)	1819 (17.9)	4255 (15.4)	
	≥12 months	708,140 (70.2)	5624 (55.3)	17,386 (62.8)	
**Oral contraceptive use, n (%)**					<.001
	None	852,165 (84.4)	7814 (76.8)	22,486 (81.2)	
	<1 year	94,738 (9.4)	1262 (12.4)	2930 (10.6)	
	≥1 year	62,511 (6.2)	1095 (10.8)	2290 (8.3)	
Age at menopause (years), mean (SD)		50.05 (4.08)	49.66 (4.27)	49.29 (4.4)	<.001
Age at menarche (years), mean (SD)		16.44 (1.87)	16.13 (1.98)	16.45 (1.94)	<.001
Total reproductive span, mean (SD)		33.6 (4.48)	33.52 (4.67)	32.84 (4.81)	<.001
**Hormone replacement therapy, n (%)**					<.001
	None	855,459 (84.8)	7803 (76.7)	22,993 (83)	
	<2 years	89,395 (8.9)	1294 (12.7)	2778 (10)	
	<5 years	36,735 (3.6)	600 (5.9)	1134 (4.1)	
	≥5 years	27,825 (2.8)	474 (4.7)	801 (2.9)	

### Effect of Smoking on Risk of Migraine

**Figure 2.**
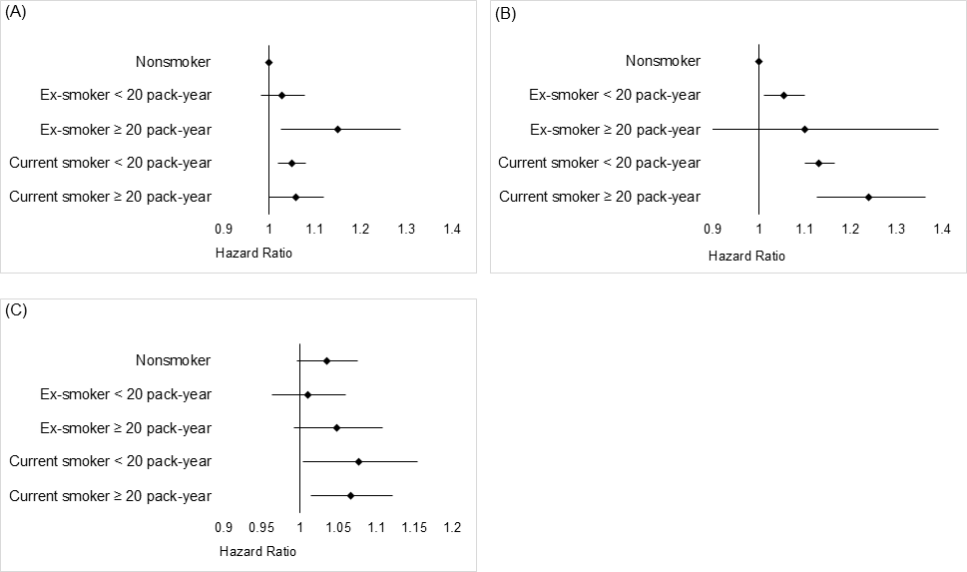
Dose-dependent relationship between smoking and the risk of migraine. (**A**) A dose-dependent relationship was observed among current smokers, indicating a higher hazard ratio as the smoking dose increased (measured in pack-years). (**B**) A dose-dependent relationship was observed for both ex-smokers and current smokers among premenopausal women, except for ex-smokers with a smoking history of ≥20 pack-years, possibly due to their relatively small sample size (n=321). (**C**) Conversely, no dose-dependent relationship was observed between smoking and migraine in postmenopausal women.

Among all participants, migraine risk was higher in both ex-smokers (adjusted HR 1.044, 95% CI 1.000‐1.089) and current smokers (adjusted HR 1.050, 95% CI 1.023‐1.079) than in nonsmokers (Table 4). In particular, a dose-dependent relationship was observed among current smokers, where those with >20 pack-years had a higher migraine risk (adjusted HR 1.058, 95% CI 1.000‐1.119) than did current smokers with <20 pack-years (adjusted HR 1.049, 95% CI 1.018‐1.080; Figure 2).

**Figure 3.**
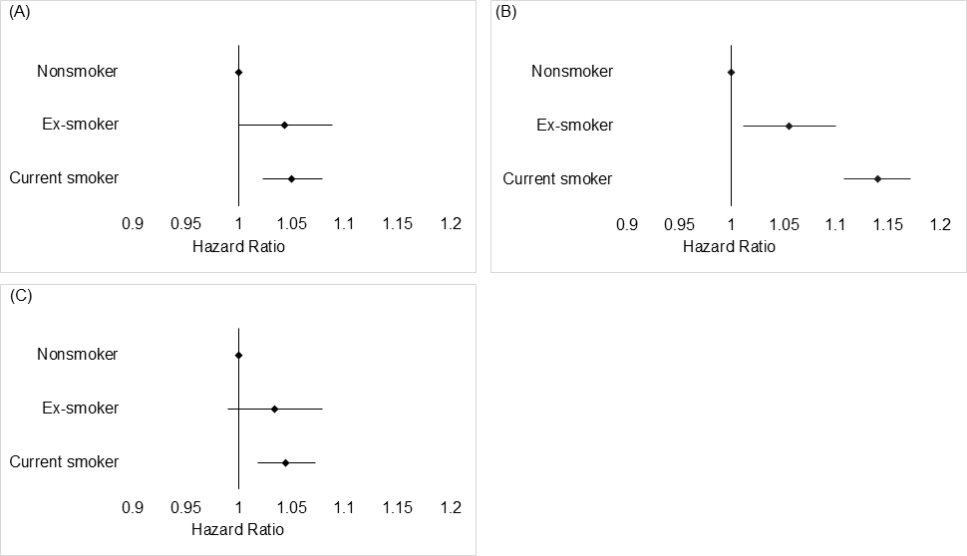
Effect of smoking on the risk of migraine. (**A**) The risk of incident migraine was higher in ex-smokers (adjusted hazard ratio [HR] 1.044, 95% CI 1‐1.089) and current smokers (adjusted HR 1.050, 95% CI 1.023‐1.079) than in nonsmokers. (**B**) The risk of incident migraine was higher in ex-smokers (adjusted HR 1.055, 95% CI 1.011‐1.1) and current smokers (adjusted HR 1.140, 95% CI 1.108‐1.172) than in nonsmokers among premenopausal women. (**C**) Among postmenopausal women, an increased risk of migraine was observed in current smokers (adjusted HR 1.045, 95% CI 1.018‐1.073) compared with nonsmokers.

In premenopausal women, the unadjusted HR was statistically significant only in current smokers (unadjusted HR 1.140, 95% CI 1.109‐1.170), revealing a greater risk of migraine than that in nonsmokers. However, after correcting for baseline imbalances and potential confounders, the data demonstrated a higher migraine risk in both ex-smokers (adjusted HR 1.055, 95% CI 1.011‐1.100) and current smokers (adjusted HR 1.140, 95% CI 1.108‐1.172) than in nonsmokers, in the multivariable model (Table 4, Figure 3). Furthermore, a dose-dependent relationship was observed in premenopausal women, showing a higher HR in relation to smoking dose (pack-years) among ex-smokers and current smokers (Figure 2). Statistical significance was not reached in ex-smokers with ≥20 pack-years of smoking history, wherein the number of participants was relatively small (n=321).

Among postmenopausal women, an increased risk of migraine was observed in current smokers (adjusted HR 1.045, 95% CI 1.018‐1.073) compared with nonsmokers (adjusted HR 1.140, 95% CI 1.108‐1.172; Table 4 and Figure 3). The effect of smoking on the development of migraine was greater, with a dose-dependent effect observed in premenopausal women compared with postmenopausal women (*P*<.001). No dose-dependent relationship was observed between smoking and migraine in postmenopausal women (Figure 2).

**Table 4.** Effect of smoking on the risk of migraine in women aged ≥40 years from the Korean health examination 2009 cohort, followed up until 2019.

Smoking status		Patients, n	Migraine	Duration	IR^a^, per 1000 PY^b^	Unadjusted, HR^c^ (95% CI)	Adjusted^d^, HR (95% CI)
**Total participants**							
	Nonsmoker	1,751,873	359,148	14,417,009	24.911	1 (Reference)	1 (Reference)
	Ex-smoker	21,674	4386	177,007	24.779	1.000 (0.958-1.044)	1.044 (1.000-1.089)
	Current smoker	53,582	11,453	430,898	26.579	1.021 (0.995-1.048)	1.050 (1.023-1.079)
	*P* value	—^e^	—	—	—	.28	< . 00 1
**Premenopausal women**							
	Nonsmoker	742,459	143,339	6,223,859	23.031	1 (Reference)	1 (Reference)
	Ex-smoker	11,503	2254	96,099	23.455	1.019 (0.977-1.062)	1.055 (1.011-1.100)
	Current smoker	25,876	5582	212,744	26.238	1.140 (1.109-1.170)	1.140 (1.108-1.172)
	*P* value	—	—	—	—	< . 001	< . 001
**Postmenopausal women**							
	Nonsmoker	1,009,414	215,809	8,193,150	26.340	1 (Reference)	1 (Reference)
	Ex-smoker	10,171	2132	80,909	26.351	1.000 (0.958-1.044)	1.034 (0.990-1.079)
	Current smoker	27,706	5871	218,155	26.912	1.021 (0.995-1.048)	1.045 (1.018-1.073)
	*P* value	—	—	—	—	.28	< .001

a IR: incidence rate.

b PY: pack-years.

c HR: hazard ratio.

d A multivariable model adjusted for age, income, BMI, alcohol consumption habits, regular exercise, diabetes mellitus, hypertension, dyslipidemia, chronic kidney disease, age at menarche, parity, breastfeeding history, and oral contraceptive use.

e Not available.

## Discussion

### Principal Results

This nationwide population-based longitudinal cohort study examined the impact of smoking on the development of migraine in women and the modifying effects of menopause. The major results of this study are the following: (1) smoking increases the risk of migraine attacks in women; (2) a dose-dependent relationship between smoking and migraine exists, particularly in premenopausal women; and (3) menopause modifies the effect of smoking on the development of migraine.

In this study, smoking increased the incidence of migraine in women. Smoking has been proposed as a potential risk factor for triggering migraine attacks. Previous cross-sectional population-based studies have consistently shown a higher prevalence of migraine among smokers [11]. Among individuals diagnosed with migraine, a higher prevalence of smoking has been observed, suggesting a potential link between smoking and the precipitation of migraine attacks [12,13]. However, other studies have shown a negative association between smoking and migraine. For instance, a population-based twin study reported no association between smoking status and migraine with aura [14]. Smoking did not have any significant influence on the risk of migraine in a population-based study conducted in Japan [15]. Similarly, a study conducted in Denmark also found no significant association between smoking and migraine, with no notable dose-response effect observed [16]. However, as these studies were cross-sectional in design, a definitive causal relationship between smoking and migraine has not yet been established. Our study addresses this knowledge gap by providing the first longitudinal data examining the potential causal relationship between smoking and migraine occurrence.

A dose-dependent relationship between smoking and migraine was observed, particularly in current smokers, with premenopausal women being more affected than postmenopausal women. This finding further supports the possible causal relationship between smoking and migraine. Various mechanisms may contribute to the association between smoking and migraine. Nicotine present in cigarettes can pass through the blood-brain barrier via active transport facilitated by the choroid plexus [21]. Further, 1 in vitro autoradiography study has shown [3H]nicotine binding receptors in various brain regions such as the interpeduncular nucleus, medial habenula, thalamic nuclei, cerebral cortex, and the dentate gyrus [22]. This was further examined through a postmortem human brain study, which revealed that smoking leads to an augmentation of high-affinity nicotine binding sites, suggesting an upregulation of nicotine receptors [23]. Additionally, trigeminal neurons play a key role in the peripheral trigeminal nociceptive system, and their involvement in developing migraine pain has been suggested [24]. In 1 study, nicotine led to a notable increase in intracellular calcium concentration in approximately half of the trigeminal neurons [25]. Chronic exposure to tobacco smoke results in neuroadaptations, including alterations in neurotransmitter systems, upregulation of nicotine receptors, and changes in neuronal excitability [26-28]. These persistent changes may contribute to long-term effects on migraine susceptibility even after smoking cessation. These findings indicate the possibility of nicotine affecting trigeminal neuron activity, which may have implications for the risk of migraine.

An interesting finding from our study was that smoking had a greater effect on migraine development in premenopausal women than in postmenopausal women, indicating that menopause modifies the impact of smoking on migraine. This suggests that smoking increases the risk of migraine more in women than in men. Estrogen level fluctuations during the menstrual cycle are associated with migraine attacks, especially during estrogen withdrawal [29-31]. Some studies indicate a decline in both the frequency and intensity of migraine episodes during menopause due to hormonal stability [32,33]. In contrast, other studies report that a portion of women experience worsening migraine after menopause [34-36]. The interplay between smoking and estrogen may impact the migraine threshold, potentially leading to migraine attacks that require hospital visits. Smoking has been shown to affect estrogen metabolism and may lead to more pronounced fluctuations in estrogen levels, potentially increasing the likelihood of migraine during hormonal shifts [37-41]. Although migraine has a genetic basis [42-44], the ability of smoking to influence estrogen levels and induce estrogen-related changes could contribute to migraine occurrence. Therefore, the interaction between smoking-induced fluctuations in estrogen metabolism and the presence of ongoing hormonal shifts in premenopausal women could result in a more pronounced detrimental effect on migraine than in postmenopausal women who experience hormonal stability. Hence, this approach could provide strong support for recommending proactive smoking cessation interventions [45-47], especially for premenopausal women, to reduce smoking-related migraine risk.

The strength of this study lies in that it is the first prospective longitudinal analysis to investigate the impact of smoking on migraine, as previous studies were limited to a cross-sectional design. The use of a large data set from the KHE and NHIS ensures a relatively unbiased population, providing valuable big data. For generalizability, we plan to investigate the total population, including both women and men, and conduct a more extensive examination of sex differences in smoking-related migraine risk. This strategic approach will allow for a better understanding of the observed differential effects between sexes and enable the exploration of potential biological explanations for these associations.

### Limitations

This study has some limitations. First, menopause was self-reported through questionnaires rather than medically confirmed. Nevertheless, self-reporting is commonly used to diagnose menopause in clinical practice and is unlikely to trigger significant errors. Second, migraine was defined using *ICD* codes. Consistent with prior studies using the NHIS database’s operational definition of migraine [48-50], the estimated prevalence of migraine code ranges between 3% and 4%, which is lower than the 1-year prevalence of migraine (6%) in the Korean population [51]. This indicates that migraine is underdiagnosed in Korea, and the operational definition of migraine in our study can be stricter than the real estimates. We believe this does not introduce significant bias to the relative risk or odds ratio estimate, as it is less prone to false positives and misclassification bias than studies whose operational definition has a potential for overdiagnosing the disease. Indeed, the underdiagnosis of migraine is a global phenomenon [5,52,53]. Although this carries the risk of an inaccurate diagnosis, it can be mitigated by a large-scale study. Finally, according to this study’s design, we defined the first insurance claim for migraine as the migraine incidence. The first insurance claim may not precisely indicate the actual onset of migraine over a lifetime because migraine is a lifetime disorder with episodic attacks, and people may not visit the hospital immediately after the first attack. Starting data analysis in 2002, due to data availability, may exclude individuals with pre-existing conditions before this timeframe and restrict the observation of certain diagnostic patterns. However, we addressed this potential limitation by implementing a washout period and accounting for the lagging effects covering approximately 10 years. Therefore, our operational definition of “migraine incidence” indicates the first hospital visit for migraine in people who did not visit a hospital in nearly 10 years. This finding suggests a plausible association between smoking and the development or worsening of migraine.

### Conclusion

Smoking increases the incidence of migraine attacks in women, and menopause modifies this effect. The interaction between smoking and estrogen may increase the vulnerability of the migraine brain.

## Abbreviations

HR: hazard ratio
*ICD-10*: *International Classification of Diseases, Tenth Revision*
KHE: Korean health examination
NHIS: National Health Insurance Service
